# Particle Swarm Optimization Algorithm for Optimizing Assignment of Blood in Blood Banking System

**DOI:** 10.1155/2015/713898

**Published:** 2015-03-01

**Authors:** Micheal O. Olusanya, Martins A. Arasomwan, Aderemi O. Adewumi

**Affiliations:** School of Mathematics, Statistics, and Computer Science, University of Kwazulu-Natal, Private Bag Box X54001, Durban 4000, South Africa

## Abstract

This paper reports the performance of particle swarm optimization (PSO) for the assignment of blood to meet patients' blood transfusion requests for blood transfusion. While the drive for blood donation lingers, there is need for effective and efficient management of available blood in blood banking systems. Moreover, inherent danger of transfusing wrong blood types to patients, unnecessary importation of blood units from external sources, and wastage of blood products due to nonusage necessitate the development of mathematical models and techniques for effective handling of blood distribution among available blood types in order to minimize wastages and importation from external sources. This gives rise to the blood assignment problem (BAP) introduced recently in literature. We propose a queue and multiple knapsack models with PSO-based solution to address this challenge. Simulation is based on sets of randomly generated data that mimic real-world population distribution of blood types. Results obtained show the efficiency of the proposed algorithm for BAP with no blood units wasted and very low importation, where necessary, from outside the blood bank. The result therefore can serve as a benchmark and basis for decision support tools for real-life deployment.

## 1. Introduction

Blood is a living tissue of unique medical value to the human body [1]. It is responsible for carrying the substances needed for healthy living to various parts of the body and carries away those not needed as wastes. A normal blood is made up of four different components, namely: red cells, white cells, platelets, and plasma [1], which serve separate functions in human organism and different use in the medical treatment of patients in the hospitals or health clinics. The need for blood every day in the hospitals for various reasons and uses in the treatment of patients has made it become of high demand. As a result, there exits collection sites where blood is collected from donors, processed, and shipped to a Hospital Blood Bank to be stored and be available to meet the demands for transfusions to patients. Handling blood transfusion in hospitals involves some complexities due to blood compatibility issues and stochastic nature of the daily demands for blood. Therefore, assignment of blood can be seen as NP-Hard problem. As a limited resource with limited shelf life, there is need for its efficient management during the process of assigning it to patients from hospitals blood banks. The component of blood considered in this paper is the red cells and it is made up of four main types, namely: A, B, AB, and O. For convenience, blood type AB will be denoted by C in this work. With the Rhesus factor [2], the number of blood types is doubled, resulting in A^+^, A^−^, B^+^, B^−^, C^+^, C^−^, O^+^, and O^−^. Optimizing the assignment of these components is the focus of this paper.

The assignment problem is one of the basic combinatorial optimization problems in the fields of discrete optimization. Many optimization techniques have been used to solve various assignment problems including vehicle assignment problem, transportation problem, task allocation problem (TAP), and blood assignment problem (BAP) [3–5]. For instance, Jean et al. [3] used PSO to solve the problem of allocating a set of cabs to some customers with the goal of minimizing the distance traveled by the fleet with result that showed that PSO is capable of achieving optimal results. Similarly, a discrete PSO (DPSO) and genetic algorithm (GA) were compared for the problems of finding optimal solution to the allocation of the expected number of people in flooded areas to various types of available vehicle in an evacuation process [6] with DPSO having better performance than GA. DPSO has also shown great performance with different degrees of difficult knapsack problems [7, 8]. In addition, experimental results in [9] showed that DPSO algorithm is highly efficient for solving the multiple knapsack problems (MKP) even with large problem instances. This motivates the investigation of DPSO in solving the BAP defined in this paper.

BAP recently stated attracting interests among metaheuristics and optimization researchers [2, 10–14] as the sourcing for blood for transfusion purpose is currently posing global challenge. An early work reported in [10] modelled the problem as a multiobjective linear programming model to help determine the best assignment of blood from donors to requests. The model was based on variables that represent the units of blood coming from both within the blood bank and outside the system and assigned available blood on daily basis depending on demand. A case study of Italian Red Cross System in Rome was considered. The model is however incapable of handling global request of different blood types at the same time. In [2] a dynamical system model was developed to handle the management of blood in a blood bank but without considering Rhesus factors. The model in [2] was improved upon in [11] where the authors modelled BAP as a MKP and compared the performances of five different (meta)heuristics in solving the problem. The goal was to minimize the amount of blood unit imported from outside the blood banking system. To further improve on the work in [11], a new cross and mutation techniques were introduced into the algorithm in [12] with the result that GA was reported as more efficient than other compared techniques. In [13], two local search optimization techniques, namely, dynamic programming and greedy randomized adaptive search procedure (GRASP) were tested for the BAP with GRASP performing better than dynamic programming. Tabu search and simulated annealing with their hybrid were also tested on BAP [14] where the hybrid showed better results than the individual techniques.

The problem of assigning blood in blood bank system principally involves attending to requests for blood and accepting donated blood from donors with the lifespan of each blood type of critical considerations. These activities are on daily basis and stochastic in nature. In situations where there is not enough volume of compatible blood type(s) to meet demands, such blood types are imported from other sources outside the blood bank system and this could be very expensive. All these put together show that the objective of assignment of blood in blood banks is to meet the daily demand for blood and to minimize the volume as well as the number of blood types imported from external source(s). Also, the volume of expired blood should be minimized. There is no report of the application of any swarm intelligence algorithms to this new domain of BAP. This paper is therefore set to investigate the application of a well-known swarm intelligence algorithm, particle swarm optimization (PSO), to the BAP.

PSO was introduced in [15, 16]. It is a fast and efficient technique used in solving complex and simple optimization problems. This technique is nature-inspired and draws inspiration from social behavior of animals, like birds and fishes. Thus, it belongs to the swarm intelligence group of evolutionary computation techniques. From the period of its inception, it has been used by researchers to solve a wide range of simple and complex optimization problems [17–24].

In the sections that follow, Section 2 gives the description of BAP considered in this paper.  Section 3 describes the method that was applied in solving the problem.  Section 4 focuses on the numerical simulations conducted which comprises the analysis and discussion of results. Finally, Section 5 concludes the paper and identifies some possible areas of future research.

## 2. Problem Description for Blood Assignment

The blood bank stores and issues the appropriate blood units to satisfy transfusion requests. On each day the bank receives a random number of transfusion requests for each blood type and each request for a random number of units. Once a request is received, the appropriate number of units of that type is removed from the bank upon successful cross-matching. We will define demand to be the number of units requested and usage to be the number of units transfused. Any units which are not used within their shelf life are considered expired and are discarded from the bank. Normally, a patient should be transfused his or her own blood type when there is a need for it. However, there could be situations when the blood type in the bank may not be enough to meet the blood units of the type requested. In order to address such challenges compatible blood types are supplied instead, where possible. The compatibility between blood types is presented in Table 1 (in this paper, blood types “C^+^” and “C^−^” refer to AB^+^ and AB^−^ throughout and are thus chosen for typo simplicity.). In the table, “YES” means that there is compatibility between the donor and receiver, while “NO” means there is no compatibility. Also, in the table, it is evident that O^−^ is the universal donor, while C^+^ is the universal receiver. In Republic of South Africa, 86% of the population has positive Rhesus factor, while 14% have negative Rhesus factor leading to the repartition presented in Table 2 [2, 11–14]. Further description of the BAP can be found in [2, 10, 12].

From the preceding description, it is evident that the assignment of blood in blood banks is a complex problem. Furthermore, it becomes more complex because blood products have limited shelf life. As a result, there is therefore the need for algorithms that could efficiently assign available blood resources in the blood bank to receivers, in order to minimize the blood units imported from external source(s) into the blood bank and at the same time minimize wastage of blood unit because of their limited shelf life.

### 2.1. Objective Function

The objective function of the BAP is given in (4). The aim is to minimize the volume of red blood cells imported into the bank over a period of *n* days:

(1) min⁡∑t=1nITotalt,

where *I*_Total_(*t*) = *I*_O^+^_(*t*) + *I*_O^−^_(*t*) + *I*_A^+^_(*t*) + *I*_A^−^_(*t*) + *I*_B^+^_(*t*) + *I*_B^−^_(*t*) + *I*_C^+^_(*t*) + *I*_C^−^_(*t*) and *I*_O^+^_, *I*_O^−^_, *I*_A^+^_, *I*_A^−^_, *I*_B^+^_, *I*_C^+^_, and *I*_C^+^_ are volumes of the respective blood types imported in day *t*.

### 2.2. Constraints

Description of different constraints that are to be satisfied in implementing the proposed algorithm to solve the BAP is as follows.

- The total volume of blood requested must be satisfied for each day, either by using the ones in the bank or by importing blood from external sources.
- The volume of blood assigned each day must not exceed the total volume available in the bank.
- The minimum volume of each blood type must not be less than zero.
- Each unit of blood types must not exceed 30 days of shelf life; otherwise, it should be incinerated.

### 2.3. Variables

Explained below are the descriptions of possible variables that can be used in the algorithm and could be represented using any meaningful variable names:

- volume of blood types in storage at day *t*;
- proportion of blood types in the population of the region considered;
- donations of blood into the bank at day *t*;
- requests for unit blood at time *t*;
- volume of incinerated expired blood at day *t*;
- volume of blood supplied at day *t*;
- volume of blood imported from external source(s) into the bank at day *t*.

### 2.4. Updating Blood Volume

On daily basis, the volumes of the blood types are updated relative to the volumes supplied, donated, and expired as stated in (15).

### 2.5. Assumptions

The model was defined with the following assumptions.

- There is sufficient supply from the external source(s) of blood, when units are required to be imported.
- The desired level of the volumes of each blood type is relative to the proportional of blood distribution in the region under consideration.
- If emergency arises, no optimization may be performed, but patients straightaway receive O^−^ blood.
- The validity date of the blood products is 30 days.

## 3. Methodology

In the daily management of the blood bank, the demand and supply of blood units vary; thus, there is a need for an effective method to determine the assignment of blood units for the purpose of good management. The assignment of blood units would be considered optimal if there is no importation of units from external sources; however, this may not be a possibility. Therefore, an algorithm that could be able to assign blood units relative to the available volume of blood units and volume requested with no or minimum units imported from outside the blood bank will suffice.

Three techniques are combined to provide solution to the BAP considered and thus to determine (near) optimal assignment of blood units relative to demands. They are particle swarm optimization which is the optimization technique used, multiple knapsack assignment technique which is used to handle the cross-matching of blood types in order to satisfy requests and stabilize the proportions of blood types stored in the bank, and queuing technique which is used to monitor the expiration date of the units of each blood type. Also described in this section is a bottom-up technique that was used to assign the blood units before importing any blood types from external source(s).

### 3.1. Particle Swarm Optimization Technique

The PSO as a population-based and stochastic technique needs a swarm of particles to carry out its optimization process [15, 16]. Using the search range defined for a problem, values are randomly generated for the decision variables. These values are then used to randomly distribute the particles in the solution search space before the technique begins its iterative process. During the optimization process, each particle communicates its new discoveries to others and this in turn determines subsequent moves of the particles in the search space. In each attempt of iteration, each particle makes use of two major pieces of information: its personal experience and the experiences of reachable neighbours to guide its search. Furthermore, the objective function of the problem being optimized is used to evaluate the quality of the discovery of each particle. Given an *n*-dimensional space, each particle is characterized by the position vector *X*_*i*_ = (*x*_*i*1_, …, *x*_*in*_) and the velocity vector *V*_*i*_ = (*v*_*i*1_, …, *v*_*in*_). When the particles are searching for optimum solution in the search space, their velocities and positions are updated using (2) and (3), respectively:

(2) Vit+1=ωVit+c1r1Pi−Xi+c2r2Pg−Xi,

(3) Xit+1=Xt+Vit+1.

In (2), *P*_*i*_ and *P*_*g*_ are vectors representing the *i*th particle personal best position and swarm global best position, respectively; *r*_1_ and *r*_2_ are random numbers in the interval [0,1], while *c*_1_ and *c*_2_ are acceleration coefficients called cognitive and social scaling parameters which determine the extent of the random forces in the direction of *P*_*i*_ and *P*_*g*_. The parameter *t* represents iteration index, while *ω* is the inertia weight which was introduced in [25]. It regulates the particle's velocity and helps to balance the global and local search abilities of PSO. In the original PSO algorithm, though not explicitly added to (2), the parameter *ω* implicitly had a value of 1. A general framework of PSO algorithm for continuous problems is presented in Algorithm 1.

Although the PSO was initially developed for continuous optimization problems, it has in several occasions been customized for discrete problems [19, 20]. Customizing PSO for discrete problems was initially proposed by Kennedy and Eberhart in [26] where they defined trajectories and velocities of particles in terms of changes of probabilities, where the particles move in a state space restricted to 0 and 1 on each dimension, with a certain probability computed using the sigmoid limiting transformation as shown in

(4) xij=1,if  μ≤Svij0,otherwise,

where *x*_*ij*_ is the value in the *j*th dimension of the position of particle *i*, *v*_*ij*_ is the value in the *j*th dimension of the velocity of particle *i*, *μ* is a value selected from a uniform distribution in [0, 1], and *S*(*v*_*ij*_) is as given in

(5) Svij=11+e−vij.

In this paper, some customization was made to the PSO algorithm before it was applied to the BAP. The different parts that were customized are as follows.

#### 3.1.1. Particle Representation

**Figure 1:**
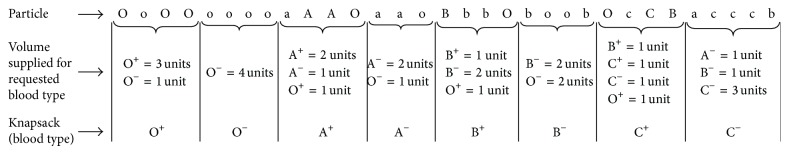
Exemplified particle representation in the proposed discrete PSO algorithm.

Each particle is encoded by a long string of characters representing each of the blood types. The string is divided into groups, with each group representing the content of each of the 8 knapsacks for each blood type. The characters in each group show which units of blood have been placed into each knapsack. Furthermore, each character represents 1 unit of positive blood type if it is uppercase (e.g., “A” for “A^+^”), but represents 1 unit of negative blood type if it is lowercase (e.g., “a” for “A^−^”). An example is shown in Figure 1.

The example in Figure 1 shows an interpretation of the representation of a typical particle in the swarm. The particle is of size (dimension) 32 and it represents the combination of all the units of requested blood types. The first 4 dimensions represent the number of units (i.e., 4) of O^+^ that was requested and the supply is made up of 3 units of O^+^ and 1 unit of O^−^. The last 5 dimensions also represent the number of units (i.e., 5) of C^−^ that was requested and the supply is made up of 1 unit of A^−^, 1 unit of B^−^, and 3 units of C^−^. The same idea of interpretation applies to the remaining dimensions of the particle. In the course of implementation of the technique, the dimension of the particles and the size of the various knapsacks vary each day relative to the changing requests received by the blood bank.

#### 3.1.2. Transformation of Particles

From Figure 1, it is clear that the values of the dimensions of the particle are alphabets. Therefore, *X*_*i*_, *P*_*i*_, and *P*_*g*_ will contain alphabets too and these cannot be directly used to compute *V*_*i*_(*t* + 1) in (2). For the velocity of each particle to be computed, these alphabets should be converted to numbers. There may be different methods to do this; however, the method used was to replace the alphabet (blood type) in each dimension of the particle with its value (see Table 4). In other words, the particle represented in Figure 1 becomes as represented in Table 3.

*P*_*i*_ and *P*_*g*_ are also transformed likewise, depending on the contents of their respective dimensions; hence, (2) can be computed to obtain a value for *V*_*i*_(*t* + 1).

#### 3.1.3. Obtaining the Positions of Particles

The value obtained for *V*_*i*_(*t* + 1) is then transformed using (5) to get a value between 0 and 1. This value is then used to determine the blood type to be imported into the blood bank when there is a shortage of blood to meet requests. In other words, the different compatible blood types (including the blood type requested) that could be used to meet the request are given equal chances to be imported from external source(s) into the blood bank. This means that if there are two compatible blood types, each is given 50% chance to be imported, if there are four compatible blood types, each is given 25% chance of being imported, and so forth. Before the beginning of next iteration, each particle is reverted back to a string of alphabets of blood types representing the new potential solution.

### 3.2. Multiple Knapsack Model

The multiple knapsack problems involve selecting any of *n* items and packing them into *m* knapsacks of different capacity *c* in order to obtain the largest sum of profit [11]. The standard model for multiple knapsack problems is stated in (6)–(9). Consider

(6)  maximize ∑i=1m∑j=1npjxij

(7)  Subject  to ∑j=1nwjxij≤ci i=1,…,m,

(8)       ∑i=1mxij≤1 j=1,…,n,

(9)       xij∈0,1 i=1,…,m;  j=1,…,n.

Equation (6) is the objective function, which is to maximize the profit of the number of items placed in all the knapsacks; (7) is the constraint that ensures that the capacity of each knapsack is not exceeded; (8) is the constraint that ensures that each item can only be placed in any of the knapsacks once and (9) indicates whether an item *j* is placed into knapsack *i* or not.

*Application of the Technique from Multiple Knapsack Problems (MKP)*. Inspired by the various successes in the application of MKP to different problems, the technique was applied to the BAP considered in this paper with some modifications. Equations (10)–(13) represent the way MKP was applied. In the case of the assignment of blood, units of blood types stored in the bank are the items, while different requests for the blood types are the knapsacks and the number of blood types compatible with each blood type is the capacity of each knapsack. Consider

(10)  minimize ∑i=1m∑j=0npjxij

(11)  Subject  to T=S+∑j=0npj

(12)       xij∈0,1 i=1,…,m;  j=1,…,n,

(13)       S,xij≥0, i=1,…,m;  j  =1,…,n,

where *T* is the total units of blood supplied from the blood bank and/or imported from external source(s), *S* is the total unit of blood supplied from the bank in each day, *p*_*j*_ is units (or volume) of blood type *j* coming from external sources, *m* is the total number of requests (number of knapsacks) to be met, and *n* is the total number of blood types available in the bank for supply (meet requests).

Equation (11) certifies that the total blood units supplied are given by the amount of blood units from the bank and external sources. Equation (12) shows whether there is a supply of blood “1” or not “0.” Equation (13) is a nonnegativity constraint; that is, all the values *S* and *x*_*ij*_ are positive integers.

In applying this technique to the BAP, some additional constraints are to be satisfied. These constraints are stated as follows.

**Figure 2:**
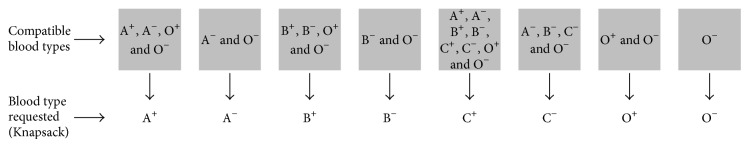
Representing blood type compatibilities as knapsack problem.

- In each day, there are 8 sets of requests for the various blood types (A^+^, A^−^, B^+^, B^−^, C^+^, C^−^, O^+^, and O^−^) which correspond to 8 knapsacks.
- To satisfy requests, units of blood within the bank or imported from external source(s) must be placed in each knapsack.
- Each knapsack can only be satisfied with a compatible unit of blood. This is illustrated in Figure 2.

It should be noted that the weight *w*_*j*_ of every unit of blood of any of the blood type is equal to 1. As a result, selecting the most profitable units that would satisfy each request in order to maximize profit is of uttermost interest.

### 3.3. Queuing Technique

**Figure 3:**
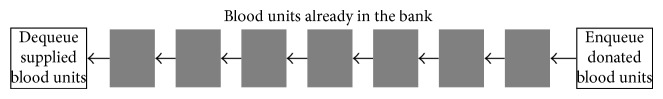
Representation of how blood units are stored in the blood bank.

In the daily management of the blood bank, it is ensured that the shelf life of each unit of blood is not exceeded in order to minimize wastage. An efficient way which can be monitored is to use the queue technique. Queue is a list-like structure that provides restricted access to its elements. Elements may only be inserted at the back (enqueue) and removed from the front (dequeue). It implements the First-In, First-Out (FIFO) operations; thus, queues release their elements in order of arrival. This means that when units of blood are supplied from the bank, they are dequeued but enqueued when units of blood are donated into the bank. This is illustrated in Figure 3, using a single-linked list. It should be noted that each of the blood type is represented separately.

Represented in (14)–(16) is the way the stack in Figure 3 is operated in the algorithm:

(14) Sj=Btot−⋃i=0rRij j=1,…,n,

(15) Btott+1=Btott∪⋃x=0dDxy y=1,…,m,

(16) Rij,Dxy≥0.

In (14) and (15), *S*_*j*_ is the total blood units of type *j* supplied by the bank, *r* is the total number of requests of all the blood types, *R*_*ij*_ is the total blood units of type *j* requested, *B*_tot_ is the total units of all the blood types in the bank, *t* represents the current day of blood bank operation, *d* is the total units of all the blood types donated, and *D*_*xy*_ is the total blood units of type *y* donated.

*Expiration of Blood Units*. As mentioned earlier, the queue is used to monitor the shelf life of each unit of the different blood type. Any of the blood units of a particular blood type that exceeds its shelf life are removed from the queue. This is represented in (16). Consider

(17) Btpt+1=Btpt−⋃i=1zEi,f, ∀f>life,

where *tp* ∈ {A^+^, A^−^, B^+^, B^−^, C^+^, C^−^, O^+^, O^−^} is the blood type, *B*_*tp*_ is the total blood units of type *tp* on queue, *t* is the current day of operation, and *E*_*i*_ is the expired blood unit *i* if its life, *f*, on the queue is greater than its shelf life, life.

### 3.4. Bottom-Up Technique

Some blood types are compatible while others are not, as shown in Figure 1. Thus, when there is a request for some units of a particular blood type, the request could be met using the same blood type if there are enough of its units in the bank. If there are not enough units (shortage) of the type requested, available units of compatible types are used to meet up the remaining units. In this case, a bottom-up approach was used in the course of assigning blood to meet the request. This means that, whenever blood is to be assigned, the requested blood type is considered first; when there is a shortage the next available compatible blood type is assigned, and so forth. Blood type O^−^ is the last option, being the universal donor; if it is not available, then blood will be imported into the bank from external source(s) relative to the request. This approach is exemplified as follows.

Given that* typ*REQ is the number of units requested of blood type* typ* and* typ*VOL is the number of units of the same blood type that is available in the bank to meet the request, if* typ*REQ >* typ*VOL, then there is a shortage of the blood type to meet the request.

## 4. Numerical Simulations

The proposed PSO algorithm proposed for solving the considered problem was investigated, while implementing the modified multiple knapsack problem, with a number of simulations with different datasets. As a result of the nonavailability of real-life data, randomly generated data were used. The stochastic nature of blood donation and requests in real-life scenario informed the usage of the fictitious randomly generated data. Whenever real-life data becomes available, they can be substituted for the fictitious data. The application software was developed in Microsoft Visual C# programming language.

### 4.1. Data Generation

All the data used were randomly generated based on the uniform random number generator. Given a specified (by the user) upper and lower bounds, random volume of requests and donation for each blood type is computed in each day. This provided the opportunity of testing the blood assignment system developed under various scenarios of varied volumes of requested and donated- blood types. Furthermore, an initial volume of all the blood types in the bank was also specified and used to calculate respective volume for each blood type using some blood type proportion. In this case, the blood type proportion in South Africa was used (see Table 1). All the parameters and their respective values as used in the algorithm to generate the testing data are presented in Table 5.

### 4.2. Parameter Setting

Presented in this section are the definitions of all the parameters used in the course of implementing the proposed model and algorithm. In Table 4, the different blood types and their respective value and proportion are presented. In Table 4, “Value” was computed based on the information in Figure 1. The numerator is the number of compatible blood types that can be received by the respective “Blood type,” while the denominator is the sum of all the compatible blood types.

For the PSO technique, the values for *c*_1_ and *c*_2_ were, respectively, set to 1.7 and parameter *ω* was set to 0.715, as recommended in [27]. The parameters *r*_1_ and *r*_2_ were randomly generated using the uniform random number generator. A swarm size of 50 particles was used and the maximum number of iteration was set to 1000.

### 4.3. Results and Discussion

**Figure 4:**
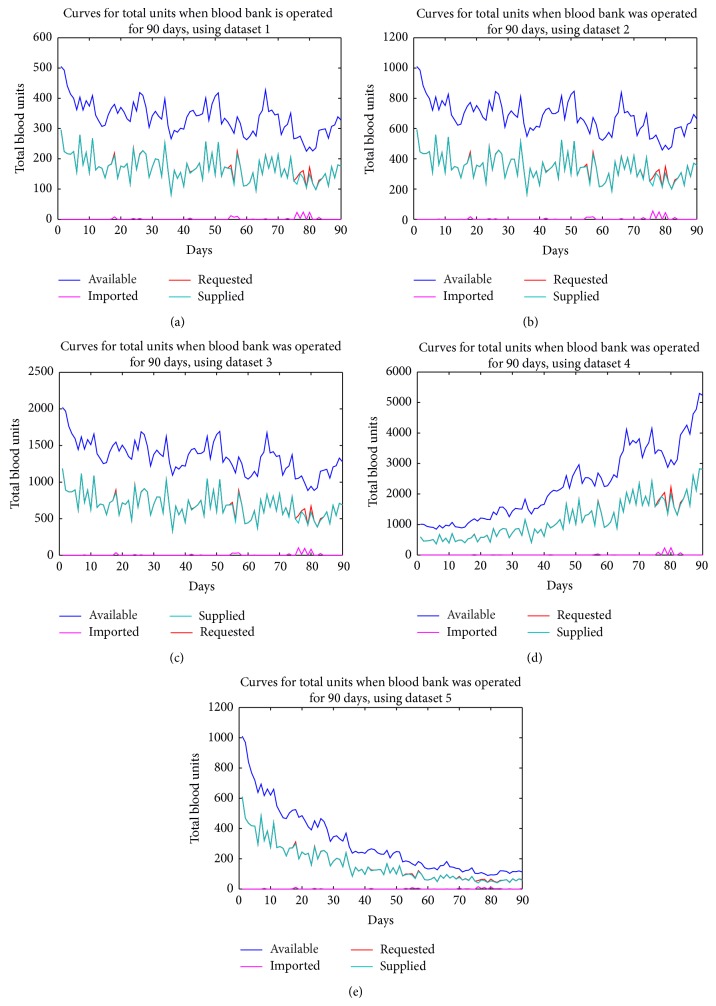
Various curves showing the different blood levels in the blood bank over 90 days, using datasets from 1 to 5.

**Figure 5:**
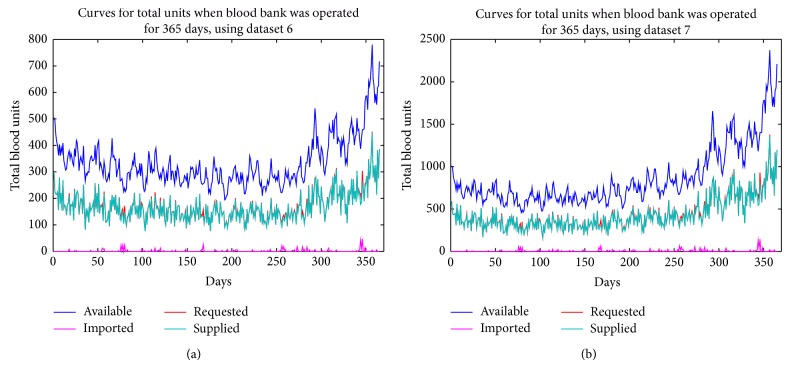
Importation rate of blood units using datasets 6 and 7.

**Figure 6:**
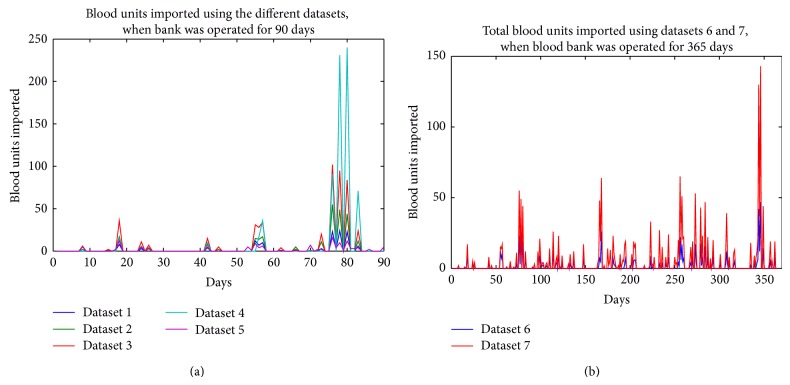
Importation rate of blood units using the different datasets.

**Figure 7:**
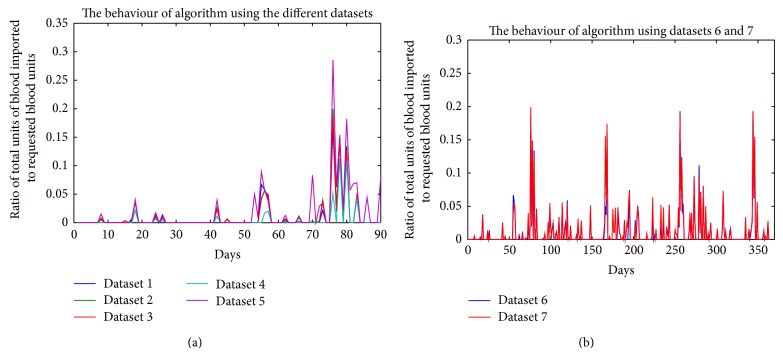
Performance measurement of the algorithm using the different datasets.

Presented in Tables 6, 7, 8, 9, 10, 11, and 12 are the average blood units that were available in, requested for, supplied by, and imported into the blood bank over the period of 90 and 365 days, using all the datasets. Figures 4–7 show the various curves associated with the management of the blood bank as observed during the simulations.

From the results, it is evident that managing the blood bank with each of the datasets over the number of specified periods (90 and 365 days) led to average importation of very few blood units from external sources. The importation became necessary when there were shortages of compatible blood types to meet requests. In Table 10, A^+^ blood type was imported into the blood bank when dataset 5 was used (with unequal ratio of requests to donations of blood units). For A^−^ blood type, apart from Table 9 (using dataset 4), some of its units were imported into the bank when the other datasets were used and the average number of units imported was in increasing order with small variations. Using any of the datasets, no units of B^+^ were imported. Some units of B^−^ were imported in Tables 6, 10, 11, and 12 (using datasets 1, 5, 6, and 7). No C^+^ blood type was imported into the bank using any of the datasets. Some units of C^−^ blood type were imported in Tables 7, 11, and 12 (using datasets 2, 6, and 7).

Much importation was observed with O^+^ and O^−^ blood types, but with O^−^, being a universal donor, the importation was more compared to others. Also, when there is a shortage of O^−^ type, no other blood types could be used as alternative(s) for it and thus it must be imported into the bank. The very low level of average importation of blood units from external source(s) is an indication that the bank was efficiently managed. This efficient management was a result of combining the efforts of the multiple knapsack problem technique used to implement the cross-matching between blood types and PSO technique used to spread importation of blood types (where necessary) in assigning blood types to meet requests. Using all the tested datasets, no blood units exceeded their shelf life; thus, no wastage of blood products was experienced. The reason for this is that the cross-matching method helps to quickly use the older blood units which have been on queue to meet requests while the new ones donated into the blood bank are enqueued.

Presented in Figures 4 and 5 are the curves showing the various volume levels of the different blood types in the blood bank when the bank was operated for 90 and 365 days, respectively. The curves show the corresponding total units of blood available, requested, supplied, and imported in each day of operating the bank. In figures, it is evident that minimal units of blood were imported from external source(s) into the bank. In the first few days no units were imported, but between 75th and 85th days a higher number of blood units were imported because the number of units requested was higher than the available blood units relative to the blood types requested. From the graphs, it appears that at the point of importation the volume of available blood in the bank was high and there could be the question, why should there be importation? The reason for importation is that the entire volume of blood in the bank comprised all the units of all blood types and the other units of blood available at the point of importation were not compatible with the blood types requested; therefore, they could not be supplied or used to meet the requests.

Also, in Figure 5, the curves show the corresponding total units of blood available, requested, supplied, and imported in each day of operating the bank for a period of 365 days. In this case, the total units of blood imported (though little) were higher than when the bank was operated for 90 days. In the graphs, it is observed that the highest units of blood were imported between the 340th and 350th days. At this period, some of the available blood types were already exhausted and there were needs for importation of blood units from external source(s) to meet the requests.

Presented in Figure 6 are the curves showing the number of blood units imported when the algorithm was tested with all the datasets. The highest importation of blood units occurred when dataset 4 was used (Figure 6(a)) and when dataset 7 was used (Figure 6(b)); these occurred around the 80th and 350th days, respectively. In Figure 7, the ratios of total units of blood imported to total units of blood requested for both 90 and 365 days of operating the blood bank are presented. For the graphs, the algorithm appears not to have performed very well with datasets 5 and 7.

## 5. Conclusion

The problem of assigning of blood units by blood banks to meet requests for blood transfusion in hospitals has been considered in this paper and an efficient method of handling this problem was proposed. The method that was proposed combined the efforts of the PSO technique and multiple knapsack problem. The multiple knapsack problem was modified to reflect some additional constraints that needed to be satisfied for the blood bank to be efficiently managed. Using the queue, multiple knapsack problem, and optimization (PSO) techniques, the total units of blood types imported into the bank were greatly minimized and no wastage was experienced.

Only the red blood cells were considered in the paper and no real-life data were used but randomly generated data. Therefore, with respect to future work, other components of the blood could be considered and the proposed method could be tested with real-life data.

## Figures and Tables

**Algorithm 1:**
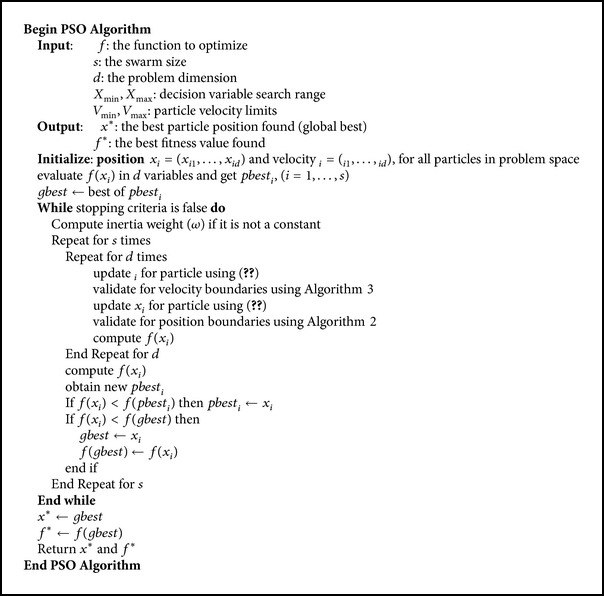


**Table 1:** Blood type compatibility with Rhesus factor.

Receiver	Donor							
	A^+^	A^−^	B^+^	B^−^	C^+^	C^−^	O^+^	O^−^
A^+^	YES	NO	NO	NO	NO	NO	YES	YES
A^−^	NO	YES	NO	NO	NO	NO	NO	YES
B^+^	NO	NO	YES	YES	NO	NO	YES	YES
B^−^	NO	NO	NO	YES	NO	NO	NO	YES
C^+^	YES	YES	YES	YES	YES	YES	YES	YES
C^−^	NO	YES	NO	YES	NO	YES	NO	YES
O^+^	NO	NO	NO	NO	NO	NO	YES	YES
O^−^	NO	NO	NO	NO	NO	NO	NO	YES

**Table 2:** Proportion of blood types in South Africa^2^.

Blood type	A^+^	A^−^	B^+^	B^−^	C^+^	C^−^	O^+^	O^−^

Proportion (%)	32	5	12	2	3	1	39	7

^2^Source: South African National Blood Service—http://www.sanbs.org.za/index.php/donors/what-s-your-type.

**Table 3:** Transformed particle used for computation in (2).

0.07	0.04	0.07	0.07	0.04	0.04	0.04	0.04	0.07	0.15	0.15	0.07	0.07	0.07	0.04	0.15	0.07	0.07	0.07	0.07	0.04	0.04	0.07	0.07	0.15	0.3	0.15	0.07	0.15	0.15	0.15	0.07

**Table 4:** Values and proportions of the different blood types.

Blood type	A^+^	A^−^	B^+^	B^−^	C^+^	C^−^	O^+^	O^−^

Value	4/27 = 0.15	2/27 = 0.07	4/27 = 0.15	2/27 = 0.07	8/27 = 0.3	4/27 = 0.15	2/27 = 0.07	1/27 = 0.04
Proportion (%)	32	5	12	2	3	1	39	7

**Table 5:** Various values used for defining the different datasets used in the simulation.

Dataset	Initial total volume of blood in bank	Running time (days)	Upper and lower bounds for requests (%)	Upper and lower bounds for donations (%)	Remark
1	500	90	[25,75]	[25,75]	These datasets were used to test the algorithm with different initial volume
2	1000	90	[25,75]	[25,75]	
3	2000	90	[25,75]	[25,75]	

4	1000	90	[25,75]	[30,75]	These datasets were used to test the algorithm when the ratios of requests to donations are unequal
5	1000	90	[30,75]	[25,75]	

6	500	365	[25,75]	[25,75]	These datasets were used to test the volume of blood units that will expire when the bank is run for a year
7	1000	365	[25,75]	[25,75]	

**Table 6:** Average volume of blood units during a runtime of 90 days with 500 blood units as initial volume (using dataset 1).

Average volume	O^+^	O^−^	A^+^	A^−^	B^+^	B^−^	C^+^	C^−^
Available in bank	114.06	17.36	109.57	19.47	41.63	8.31	21.02	4.96
Requested	66.79	11.87	54.43	8.41	19.87	3.31	5.08	1.77
Supplied	65.33	11.97	54.32	8.49	19.87	3.42	5.08	1.57
Imported into bank	0.42	1.03	0.00	0.01	0.00	0.01	0.00	0.00

**Table 7:** Average volume of blood units during a runtime of 90 days with 1000 blood units as initial volume (using dataset 2).

Average volume	O^+^	O^−^	A^+^	A^−^	B^+^	B^−^	C^+^	C^−^
Available in bank	231.34	34.59	216.72	40.36	80.79	12.56	45.67	15.77
Requested	134.59	23.87	109.73	16.88	40.10	6.68	10.23	3.31
Supplied	131.66	24.19	109.47	17.09	39.96	6.81	10.23	2.97
Imported into bank	0.90	2.08	0.00	0.02	0.00	0.00	0.00	0.02

**Table 8:** Average volume of blood units during a runtime of 90 days with 2000 blood units as initial volume (using dataset 3).

Average volume	O^+^	O^−^	A^+^	A^−^	B^+^	B^−^	C^+^	C^−^
Available in bank	460.20	69.17	430.09	80.11	159.83	26.31	90.77	30.54
Requested	267.53	47.46	218.10	33.68	79.71	13.24	20.42	6.54
Supplied	261.86	48.09	217.62	33.91	79.47	13.67	20.42	5.90
Imported into bank	1.69	4.01	0.00	0.06	0.00	0.00	0.00	0.00

**Table 9:** Average volume of blood units during a runtime of 90 days with 1000 blood units as initial volume (using dataset 4).

Average volume	O^+^	O^−^	A^+^	A^−^	B^+^	B^−^	C^+^	C^−^
Available in bank	759.21	126.86	773.10	146.51	260.02	46.24	112.67	62.60
Requested	449.09	80.66	372.94	56.67	135.39	22.91	34.90	10.08
Supplied	439.63	82.37	372.94	56.63	135.39	23.17	34.90	9.59
Imported into bank	2.29	5.72	0.00	0.00	0.00	0.00	0.00	0.00

**Table 10:** Average volume of blood units during a runtime of 90 days with 1000 blood units as initial volume (using dataset 5).

Average volume	O^+^	O^−^	A^+^	A^−^	B^+^	B^−^	C^+^	C^−^
Available in bank	108.80	15.39	82.01	14.78	41.89	6.63	29.31	6.51
Requested	64.07	11.33	51.89	8.11	18.93	3.12	4.79	1.69
Supplied	63.24	11.24	51.52	8.07	18.84	3.28	4.79	1.50
Imported into bank	0.33	0.91	0.02	0.17	0.00	0.01	0.00	0.00

**Table 11:** Average volume of blood units during a runtime of 365 days with 500 blood units as initial volume (using dataset 6).

Average volume	O^+^	O^−^	A^+^	A^−^	B^+^	B^−^	C^+^	C^−^
Available in bank	116.71	16.04	88.33	14.69	54.68	6.43	28.35	10.55
Requested	66.05	11.79	53.84	8.37	20.01	3.36	5.07	1.72
Supplied	65.27	11.77	53.04	8.38	19.89	3.37	5.07	1.61
Imported into bank	0.41	1.03	0.00	0.30	0.00	0.05	0.00	0.01

**Table 12:** Average volume of blood units during a runtime of 365 days with 1000 blood units as initial volume (using dataset 7).

Average volume	O^+^	O^−^	A^+^	A^−^	B^+^	B^−^	C^+^	C^−^
Available in bank	286.50	39.22	220.99	35.68	140.24	14.68	80.41	43.34
Requested	169.22	30.23	137.99	21.49	51.36	8.65	12.93	4.32
Supplied	166.98	30.05	135.69	21.36	51.05	8.58	12.93	4.09
Imported into bank	1.22	3.08	0.00	0.98	0.00	0.17	0.00	0.01
